# Giant Mediastinal Germ Cell Tumour: An Enigma of Surgical Consideration

**DOI:** 10.1155/2016/7615029

**Published:** 2016-10-11

**Authors:** Firdaus Hayati, Nurayub Mohd Ali, Levin Kesu Belani, Nornazirah Azizan, Andee Dzulkarnaen Zakaria, Mohd Ramzisham Abdul Rahman

**Affiliations:** ^1^Department of Surgery, Faculty of Medicine and Health Sciences, Universiti Malaysia Sabah, Kota Kinabalu, Sabah, Malaysia; ^2^Department of Surgery, Universiti Kebangsaan Malaysia Medical Centre, Kuala Lumpur, Malaysia; ^3^Department of Pathobiology and Medical Diagnostic, Faculty of Medicine and Health Sciences, Universiti Malaysia Sabah, Kota Kinabalu, Sabah, Malaysia; ^4^Department of Surgery, School of Medical Sciences, Universiti Sains Malaysia, Kota Bharu, Kelantan, Malaysia

## Abstract

We present a case of 16-year-old male, who was referred from private centre for dyspnoea, fatigue, and orthopnea. The chest radiograph revealed complete opacification of left chest which was confirmed by computed tomography as a large left mediastinal mass measuring 14 × 15 × 18 cm. The diagnostic needle core biopsy revealed mixed germ cell tumour with possible combination of embryonal carcinoma, yolk sac, and teratoma. After 4 cycles of neoadjuvant BEP regime, there was initial response of tumour markers but not tumour bulk. Instead of classic median sternotomy or clamshell incision, posterolateral approach with piecemeal manner was chosen. Histology confirmed mixed germ cell tumour with residual teratomatous component without yolk sac or embryonal carcinoma component. Weighing 3.5 kg, it is one of the largest mediastinal germ cell tumours ever reported. We describe this rare and gigantic intrathoracic tumour and discuss the spectrum of surgical approach and treatment of this exceptional tumour.

## 1. Introduction

Germ cell tumours are embryologically derived from reproductive cells, and they originate mostly from the gonads. However, in 5% of the cases, they are extragonadal in origin [1]. The most common extragonadal site is reported to be in the mediastinum [2]. Primary mediastinal nonseminomatous germ cell tumour is a rare entity, and it accounts for 5% of all germ cell tumours [3].

The presentations vary ranging from accidental findings on routine radiography to life-threatening respiratory and cardiovascular compromise. Huge intrathoracic mass poses a dramatic challenge for operating surgeons and anaesthetists in terms of the management strategies. According to a surgeon's point of view, the nature of mass suggests the possible surgical difficulties with regard to the approach and accessibility. A huge intrathoracic mass may compress the contralateral lung during positioning which may obstruct the venous return to the heart and thus poses a challenge to the attending anaesthetist.

We present a case of gigantic intrathoracic germ cell tumour which was resected successfully via a piecemeal surgical approach. The anatomical basis of this huge tumour and the treatment modalities are discussed.

## 2. Case Report

A previously well 16-year-old male was referred from a private hospital to our tertiary medical centre with acute history of dyspnoea, fatigue, and orthopnea. He denied history of fever, pleuritic chest pain, dysphagia, and loss of weight. Clinically, there was stony dullness on the left chest wall. No abnormalities were detected in other systemic examinations.

**Figure 1:**
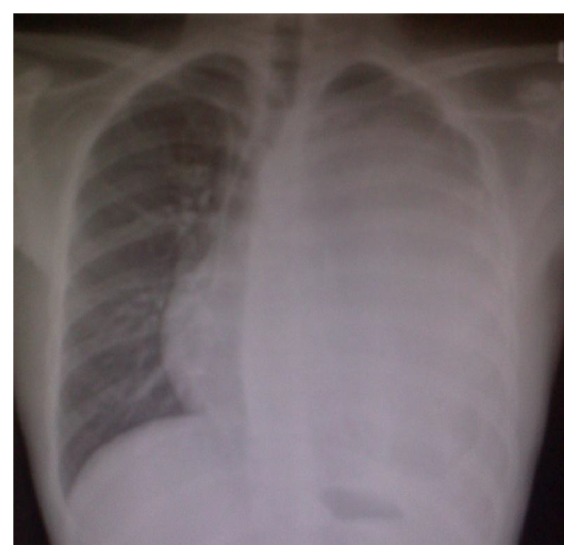
Chest radiograph showing left mediastinum mass.

**Figure 2:**
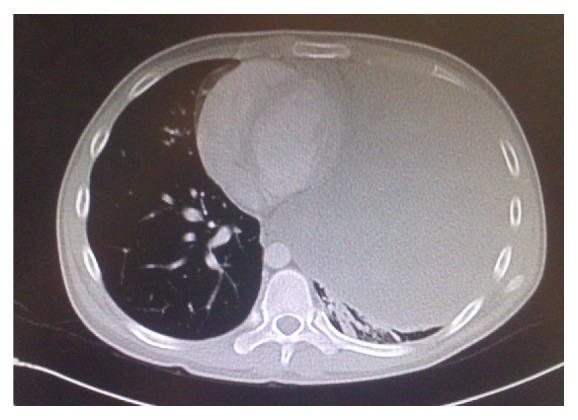
Anteroposterior view of CT scan showing that the mass occupies the whole of the left thoracic space.

**Figure 3:**
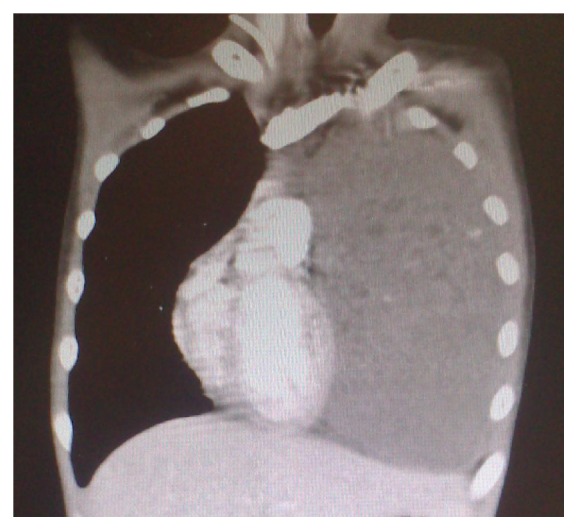
Coronal view of the CT scan showing that the mass occupies the whole of the left thoracic space with mediastinal shift to the right.

Chest radiograph showed a generalized haziness of left chest (Figure 1). Computed tomography (CT) of thorax revealed a large left mediastinal mass size measuring 14 × 15 × 18 cm (Figures 2 and 3). His baseline tumour markers showed alpha fetoprotein (AFP) level of 36920 ng/mL [normal value: <5 ng/mL] and lactate dehydrogenase (LDH) level of 893 iu/L [normal value: 140–333 iu/L]. CT-guided biopsy was performed which was suggestive of mixed germ cell tumour with possible combination of embryonal carcinoma, yolk sac, and teratoma.

He was given standard neoadjuvant chemotherapy consisting of bleomycin, etoposide, and cisplatin-based chemotherapy (BEP) regime for 4 cycles. Tumour markers after chemotherapy improved remarkably with AFP of 17 ng/mL and LDH of 477 iu/L. Unfortunately, beta human chorionic gonadotropin (beta-HCG) was not assessed during the course of chemotherapy. Despite biochemical improvement, there was no tumour reduction upon reassessment of CT scan.

**Figure 4:**
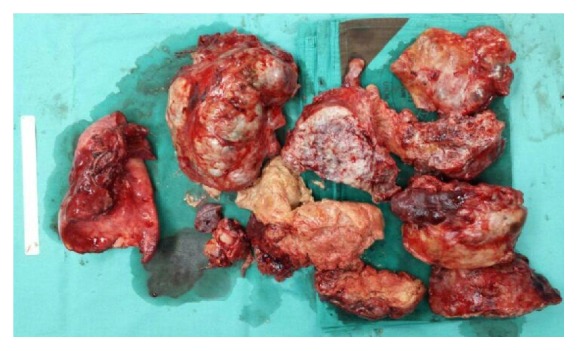
Photograph showing tumour removal via piecemeal approach.

He was decided for tumour debulking to reduce the tumour load. Patient was put on general anesthesia with double lumen ventilation. A standard left posterolateral skin incision was made. In order to achieve minimal incision, the tumour was dissected via piecemeal manner (Figure 4). Intraoperatively, the tumour was found to compress the left lung causing difficulty to differentiate tumour tissue and lung parenchyma, and hence decision to perform pneumonectomy was decided. The surgery went well without any complications.

Postoperative recovery was uneventful. Assisted ventilation was withdrawn 12 hours after operation. The tumour weighed 3.5 kg. Histopathologic evaluation revealed mixed germ cell tumour with residual teratomatous component. There was no yolk sac or embryonal carcinoma component seen. However, the lung tissue was firmly adhered to the tumour but no obvious tissue infiltration. He was discharged after one week following hospitalization without any postoperative complication. Currently, he is under oncology follow-up for further management.

## 3. Discussion

Germ cell tumours are embryologically derived from reproductive cells. In majority, they are originated from gonadal organs. It is unusual to find germ cell tumours which are extragonadal in origin, whereby it accounts for 5% of the cases [2]. The most common extragonadal sites include mediastinum, retroperitonium, vagina, and brain [4]. They have been also reported at sites such as lung, liver, prostate, and omentum [4]. Conventional anatomy textbooks do not highlight the abnormal sites of germ cell tumours, hence giving the case reports as the only source of information. Researchers suggested that there is abnormal cell migration during embryogenesis or profuse distribution of germ cells to organs such as liver, thymus, bone marrow, and brain [5]. These cells act with different regulatory function at sites mentioned above or transmit valuable genetic, hematologic, or immunologic information [5].

The clinical features vary differently from accidental findings on routine radiography to life-threatening respiratory and cardiovascular compromise. Symptoms arising from such huge tumours are due to compressive effect on the surrounding organs which include cough, shortness of breath, failure symptoms, and chest pain or due to tumour rupture such as pleural effusion and pericardial effusion [6, 7]. The largest ever mixed germ cell tumour of the mediastinum reported was 21 × 20 × 16 cm in size and weighed 3 kg, thus giving this present case a bizarre literature especially originating from South-East Asia [8].

Preoperative biopsy is indicated in this case in order to guide our treatment strategy. The role of clinical assessment is highly limited. Hence, radiological finding to determine the tumour origin and location in the mediastinum is crucial. Fine needle core biopsy is accepted as the standard procedure for confirmatory histological diagnosis [1]. It should be performed under radiologic guidance by well-trained personals due to extensive vital structures surrounding it. It is essential as lymphoma was one of the differential diagnoses, guided by an elevated LDH level biochemically. Standard treatment for primary mediastinal lymphoma is 6 cycles of chemotherapy alone, namely, R-CHOP (rituximab, cyclophosphamide, doxorubicin, vincristine, and prednisone) protocol, and hence by diagnosing it avoids unnecessary mutilating surgery.

Tumour markers are frequently elevated in germ cell tumours, namely, LDH, AFP, and beta-HCG [9]. Tumour marker measurement is mandatory in assessing the response to chemotherapy especially in chemosensitive GCT. Raised serum AFP levels indicate the presence of yolk sac and embryonal elements in mixed germ cell tumours, as seen in our case. This makes a GCT more likely to be the provisional diagnosis. A rapid decline of tumour marker levels after platin-based chemotherapy is associated with improved overall survival.

**Figure 5:**
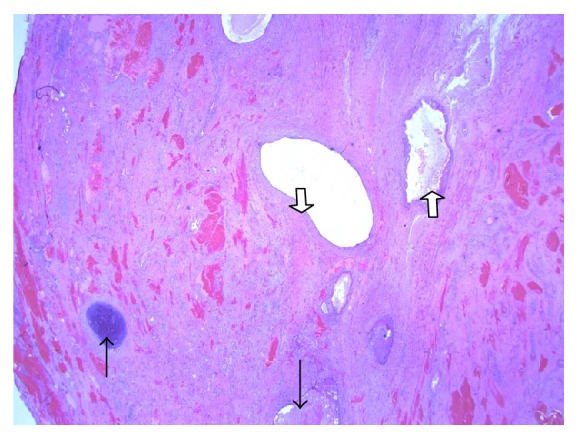
Photograph showing histological features of teratomatous components. Upward black head arrow: mature cartilage. Upward white arrow: respiratory epithelium. Downward black head arrow: squamous epithelium with keratin cyst. Downward white arrow: columnar epithelium with goblet cells.

Almost 70% of the nonseminomatous germ cell tumours contain more than two germ cell components, so they are termed as mixed germ cell tumours [10]. The main components of the mixed type germ cell tumour are yolk sac tumour and teratoma [11]. In this reported case, the histopathological examination revealed nonseminomatous mixed germ cell tumour with three germ cell components, namely, teratoma, embryonal carcinoma, and yolk sac. The teratoma component is composed of islands of mature stratified squamous epithelium, keratin cyst formation, mature cartilages, clusters of columnar epithelium with goblet cells, and cystic areas lined by mature ciliated respiratory epithelium (Figure 5). Immunohistochemical studies highlighted the presence of embryonal and yolk sac tumour components as evidenced by positive CD30 and AFP, respectively. However, beta-HCG for choriocarcinoma and PLAP for seminoma were negative.

In the era of minimally invasive surgery, small mediastinal tumours have been surgically removed via laparoscopic procedure, but large masses are best managed using median sternotomy approach [12]. A clamshell incision provides the best exposure for surgical handling in comparison to median sternotomy when it comes to extremely huge tumours. It is not without its shortcomings as it only provides bilateral exposure and does not provide vertical dimension handling. Thus, using a lateral sternal split with anterior thoracotomy at the level of the third intercostal space may provide a better alternative.

According to the observed reason, we have decided to perform alternative method which could provide more benefit to the patient. Using standard posterolateral incision, access was obtained. The patient was placed in lateral decubitus position with padding to the elbows and knees. The lower hand was put straight on arm board and the upper hand was rotated in a forward direction and allowed to hang over the operating table. This position allowed free rotation of the scapula. The inferior angles of the scapula, spinal, and axillary borders were identified. The incision was made from a point located 3 inches from midspine vertebral line to the anterior axillary line, passing below the tip of the scapula. The incision was made and deepened until fascia overlying the latissimus dorsi and trapezius muscles. Latissimus dorsi was transected sparing the trapezius muscle. The serratus anterior was retracted anteriorly to get access to the rib cage. Selection of the appropriate intercostal space was guided by counting the ribs whereby the hand slipped below the scapula and was gently pushed upwards to the apex.

The 6th intercostal space was identified and the pleural space was entered after division of the intercostal muscles with the electrocautery probe. The dissection was near to the lower rib of the interspace to avoid injury to the neurovascular bundle. A large rib spreader was inserted and opened slowly and progressively to minimize the risk of rib fracture. The tumour was dissected via piecemeal manner rather than* en bloc* dissection. Complete excision of the mass was challenging as hugeness of the tumour compressing on the left lung. Besides, it caused difficulty to differentiate between tumour tissue and lung parenchyma; hence decision to perform pneumonectomy was decided. The surgery was performed with a double lumen endotracheal tube in place. Adequate deflation of the underlying lung was in order to facilitate exposure of the surgical field and to avoid risking of the contralateral lung. Such method is favourable as it enhances patient's recovery postoperatively besides having perfect cosmetic results. Complications of surgery include bleeding, pyothorax, and phrenic nerve injury.

Primary mediastinal nonseminomatous germ cell tumours are considered as poor risk status and guarded prognosis with a 5-year survival rate of 48% [13, 14]. Chemotherapy is the mainstay of initial treatment and surgery should be viewed as an adjuvant to chemotherapy. BEP regime is the current standard for poor risk status, given for 4 cycles [13]. Partial response or residual masses with normalised AFP levels are subjected for surgical resection [9]. Residual embryonal, yolk sac, choriocarcinoma, or seminoma elements upon final HPE need second-line chemotherapy of another 2 cycles [13]. Upon being discharged, he was being followed up only by the oncologist as no yolk sac or embryonal carcinoma component is seen from the final HPE.

Even if the patient initially presented with acute respiratory distress, we had decided to offer him BEP regime in view of age factor and good performance status. The usage of bleomycin can lead to progressive pulmonary fibrosis. In certain centre, 4 cycles of VIP (etoposide, ifosfamide, and cisplatin) regime are used for those who may not tolerate bleomycin. Unfortunately, ifosfamide is not available in our centre. Since he has a potential respiratory complication, intraoperative lung care was carried out aggressively. High concentration of supplemental oxygen was avoided because of the potential harmful effects after neoadjuvant treatment with bleomycin [15].

Other prognostic factors include nonpulmonary visceral metastases and presence of high tumour markers (beta-HCG > 50000, AFP > 10000, and LDH > 10x upper limit of normal) [8]. Besides, there are several independent factors that predict poor survival include persistent germ cell tumour in residual mass, sarcoma degeneration, and postchemotherapy AFP level greater than 1001 ng/mL [16]. To date, radiotherapy has not proven to be sensitive for the treatment of primary mediastinal nonseminomatous germ cell tumours [17].

## 4. Conclusion

Primary mediastinal nonseminomatous germ cell tumours are rare in origin. Following chemotherapy, despite regression of the tumour biochemically and histologically, the size remains the same and requires surgical intervention. The present case was a humble attempt to highlight the extragonadal location of the germ cell tumour, its clinical features, and treatment which may be beneficial for academicians and clinicians.
